# High Expression Levels of CDK1 and CDC20 in Patients With Lung Squamous Cell Carcinoma are Associated With Worse Prognosis

**DOI:** 10.3389/fmolb.2021.653805

**Published:** 2021-07-07

**Authors:** Huan Deng, Qingqing Hang, Dijian Shen, Hangjie Ying, Yibi Zhang, Xu Qian, Ming Chen

**Affiliations:** ^1^ Cancer Hospital of the University of Chinese Academy of Sciences (Zhejiang Cancer Hospital), Hangzhou, China; ^2^ Institute of Cancer Research and Basic Medical (IBMC), Chinese Academy of Sciences, Hangzhou, China; ^3^ Department of Radiation Oncology, Zhejiang Key Laboratory of Radiation Oncology, Zhejiang Cancer Hospital, Hangzhou, China; ^4^ College of Life Sciences, University of Chinese Academy of Sciences, Beijing, China; ^5^ The Second Clinical Medical College, Zhejiang Chinese Medical University, Hangzhou, China; ^6^ Jiangxi Medical College, Nanchang University, Nanchang, China

**Keywords:** lung squamous cell carcinoma, protein-protein interaction network, survival analysis, gene set enrichment analysis, experimental validation

## Abstract

**Purpose:** Progress related to the early detection and molecular targeted therapy of lung squamous cell carcinoma (LUSC) remains limited. The goal of our study was to identify key candidate indicators of LUSC.

**Methods:** Three microarray datasets (GSE33532, GSE30219 and GSE19188) were applied to find differentially expressed genes (DEGs). Functional enrichment analyses of DEGs were carried out, and their protein-protein interaction (PPI) network was established. Hub genes were chosen from the PPI network according to their degree scores. Then, overall survival (OS) analyses of hub genes were carried out using Kaplan-Meier plotter, and their GSEA analyses were performed. Public databases were used to verify the expression patterns of CDK1 and CDC20. Furthermore, basic experiments were performed to verify our findings.

**Results:** A total of 1,366 DEGs were identified, containing 669 downregulated and 697 upregulated DEGs. These DEGs were primarily enriched in cell cycle, chromosome centromeric region and nuclear division. Seventeen hub genes were selected from PPI network. Survival analyses demonstrated that CDK1 and CDC20 were closely associated with OS. GSEA analyses revealed that cell cycle, DNA replication, and mismatch repair were associated with CDK1 expression, while spliceosome, RNA degradation and cell cycle were correlated with CDC20 expression. Based on The Cancer Genome Atlas (TCGA) and The Human Protein Atlas (THPA) databases, CDK1 and CDC20 were upregulated in LUSC at the mRNA and protein levels. Moreover, basic experiments also supported the obvious upregulation of CDK1 and CDC20 in LUSC.

**Conclusion:** Our study suggests and validates that CDK1 and CDC20 are potential therapeutic targets and prognostic biomarkers of LUSC.

## Background

Lung cancer is one of the most common cancer types with high cancer-related mortality and is estimated to lead to 228,820 cancer cases and 135,720 deaths in 2020 (Siegel et al., 2020). Non-small cell lung cancer (NSCLC) accounts for 85% of total lung cancer cases, and lung adenocarcinoma (LUAD) is the first prevalent histological type of NSCLC followed by lung squamous cell carcinoma (LUSC). Accumulating evidence indicated that overexpression and mutation of the genes are associated with carcinogenesis, proliferation, and/or worse prognosis of LUSC, including phosphatidylinositol-4,5-bisphosphate 3-kinase catalytic subunit alpha (PIK3CA), MET proto-oncogene (MET), and epidermal growth factor receptor (EGFR) (Sands et al., 2020). Numerous studies have demonstrated considerable progress of molecular targeted therapy for LUAD, including tyrosine kinase inhibitors targeting EGFR and anaplastic lymphoma kinase (ALK) (Li et al., 2016). However, the progress in the diagnosis and treatments of LUSC is extremely limited compared with that in LUAD. Thus, key biomarkers involved in the carcinogenesis and progression of LUSC need to be identified, and effective therapeutic measures need to be developed.

In the last 2 decades, gene chip technologies and bioinformatics analysis have been rapidly developed for the screening of genetic alterations at the genome level (Dawany et al., 2011). These technologies are used to identify differentially expressed genes (DEGs), which play important roles in the development of LUSC. However, false positive rates of independent microarray studies may influence the reliability of the outcomes (Manoli et al., 2006).

Therefore, in the current study, we selected three microarray datasets from Gene Expression Omnibus (GEO) database to identify DEGs in LUSC and normal lung tissues. Functional enrichment analyses and protein-protein interaction (PPI) network analysis were carried out to determine the molecular mechanisms of initiation and invasion of LUSC. The top 17 genes with the highest degree scores were selected as hub genes of the PPI network. CDK1 and CDC20 were survival-related hub genes. GSEA analyses of CDK1 and CDC20 were performed. Finally, our findings are validated in public databases and basic experiments.

## Materials and Methods

### The Obtainment of Microarray Datasets

GEO (Barrett et al., 2013) is the public functional genomics data repository containing the results of high throughput gene expression, chips, and microarrays. All microarray datasets were selected according to the following selection criteria: 1) the inclusion of LUSC and normal lung tissue samples; 2) the platforms were GPL570 [HG-U133_Plus_2] Affymetrix human genome U133 plus 2.0 array; 3) the organisms were *Homo sapiens*; and 4) the size of the normal lung tissue samples was >3. Three gene expression profiles (GSE33532 (Meister et al., 2014), GSE30219 (Rousseaux et al., 2013), and GSE19188 (Hou et al., 2010)) were downloaded from GEO (Table 1).

**TABLE 1:** Characteristics of the included datasets from GEO.

GEO datasets	Publication year	Country	RNA-Seq platforms	LUSC	Normal	Sum
GSE33532 (Meister et al., 2014)	2014	Germany	GPL570	16	20	36
GSE30219 (Rousseaux et al., 2013)	2013	France	GPL570	61	14	75
GSE19188 (Hou et al., 2010)	2010	Netherlands	GPL570	27	65	92

Abbreviations: GEO, gene expression omnibus; LUSC, lung squamous cell carcinoma.

### Identification of Differentially Expressed Genes

DEGs were selected between LUSC and normal lung tissues using the limma package of R software (Ritchie et al., 2015). To minimize the false discovery rate (FDR), the *p*-values were adjusted using the Benjamini–Hochberg method. Probe sets without corresponding gene symbols were omitted, while genes with >1 probe sets were averaged. The filtering criteria for DEGs were adjusted *p* < 0.05 and |logFC(fold change)|>1.

### Functional Enrichment Analysis of DEGs

To analyze DEGs’ biological functions, gene ontology (GO) and kyoto encyclopedia of genes and genomes (KEGG) enrichment analyses were carried out through the clusterProfiler (Yu et al., 2012) and GOplot packages. GO is an important bioinformatics tool for gene annotation and the analysis of biological processes of genes (Ashburner et al., 2000). Moreover, Kyoto Encyclopedia of Genes and Genomes (KEGG) is used to identify high-level functions and biological systems based on the large-scale molecular datasets (Kanehisa et al., 2017). Adjusted *p* < 0.05 was regarded as statistically significant.

### Construction of the PPI Network and Selection of Hub Genes

The PPI network of DEGs was constructed using Search Tool for the Retrieval of Interacting Genes (STRING) (Franceschini et al., 2013), and the interaction with a combined score>0.9 was regarded as statistically different. Cytoscape is a bioinformatics software that is used to establish visual networks of molecular interactions (Smoot et al., 2011). Significant gene modules were visualized using The Molecular Complex Detection (MCODE) plugin which was used to identify closely correlated modules from PPI networks (Bandettini et al., 2012). The criteria of selection were MCODE score>5, node score cutoff = 0.2, degree cutoff = 2, k-score = 2, and max depth = 100.

Hub genes were chosen based on the degree scores using the cytoHubba plugin (Chin et al., 2014). The GO enrichment and KEGG pathway analysis of the hub genes were carried out using the ClueGO plugin (Mlecnik et al., 2019). We still explored the correlation network of these hub genes. Based on The Cancer Genome Atlas (TCGA)-LUSC dataset, heatmap of these hub genes was constructed and visualized using pheatmap package. The Kaplan-Meier (KM) plotter was applied to explore the prognostic values of hub genes among LUSC patients (Győrffy et al., 2013). Log-rank *p* < 0.05 was considered statistically different.

### GSEA Analysis of the Hub Genes

GSEA is a computational method which estimates whether a previously defined gene set has statistical differences and concordant differences in two biological states (Subramanian et al., 2005). We divided LUSC samples into high-expression and low-expression groups based on the median expression levels of CDK1 and CDC20. The effects of the expression of CDK1 and CDC20 on multiple gene sets were analyzed in the relevant KEGG pathways using the molecular signatures database (MSigDB) (c2. cp.kegg.all.v7.1. symbols.gmt) (Liberzon et al., 2011). The permutation of each analysis was 1,000 times. |Normalized enrichment score (NES)|> 1, NOM *p*-value<0.05, and FDR *q*-value <0.25 were used to identify significant differences.

### The Verification Based on Public Databases

To compare the mRNA expression levels of CDK1 and CDC20 between LUSC and normal lung tissues, the expression patterns of the two genes were explored using the TCGA-LUSC dataset. Additionally, we assessed whether the expression levels of the two genes are associated with clinical information. To further explore their protein expression patterns, the immunohistochemistry (IHC) outcomes of the two genes were compared between the normal and LUSC tissue samples using The Human Protein Altas (THPA) database.

### The External Validation of Basic Experiments

20 pairs of LUSC and normal lung tissues were collected from Zhejiang Cancer Hospital (Zhejiang, China) from 2015 to 2020 (Supplementary Table 1). These tissues were frozen in liquid nitrogen for further analysis, containing real time-quantitative PCR (RT-qPCR), western blot and IHC. Besides, these experiments were independently repeated in triplicate. These experiments obtained the approval of the Medical Ethics Committee of Zhejiang Cancer Hospital (IRB-2020-817).

Firstly, total RNA extracted from these tissues using Trizol Reagent (TaKaRa, Japan) were reverse transcribed with RT reagent Kit gDNA Eraser (TaKaRa, Japan). SYBR-Green (TaKaRa, Japan) and RT-qPCR analysis were applied to detect cDNA expression levels, and GAPDH was applied as the internal reference. Primers of GAPDH, CDK1 and CDC20 in RT-qPCR were displayed (Table 2).

**TABLE 2:** The primers of GAPDH, CDK1 and CDC20.

Gene symbol	Forward (F)	Reverse (R)
GAPDH	5′-ACA​ACT​TTG​GTA​TCG​TGG​AAG​G-3′	5′-GCC​ATC​ACG​CCA​CAG​TTT​C-3′
CDK1	5′-GGA​TGT​GCT​TAT​GCA​GGA​TTC​C-3′	5′-CAT​GTA​CTG​ACC​AGG​AGG​GAT​AG-3′
CDC20	5′-GAC​CAC​TCC​TAG​CAA​ACC​TGG-3′	5′-GGG​CGT​CTG​GCT​GTT​TTC​A-3′

Secondly, total proteins were extracted using lysis buffer including protease inhibitors, and the concentrations of proteins were measured. PVDF membrane (Millipore, Bedford, MA) containing proteins separated on SDS-PAGE gels was blocked using 5% BSA and incubated using primary antibodies. Immunoblot was discovered using ECL detection reagent (Millipore). Antibodies used were as follows: CDK1 (Abcam, ab18), CDC20 (Abcam, ab155921), and GAPDH (Abcam, ab8245). The expressions of CDK1 and CDC20 were normalized against GAPDH expression.

Thirdly, the sections of these tissues via deparaffinization and dehydration were incubated with anti-CDK1 (dilution: 1:100) and anti-CDC20 antibodies (dilution: 1:300) overnight at 4°C after epitope retrieval, hydrogen peroxide treatment and non-specific antigens blocking. Then, these sections were incubated using secondary antibodies, followed by signal detection with the DAB staining kit (Vector Laboratories, United States).

## Results

### Identification of DEGs in LUSC

**FIGURE 1:**
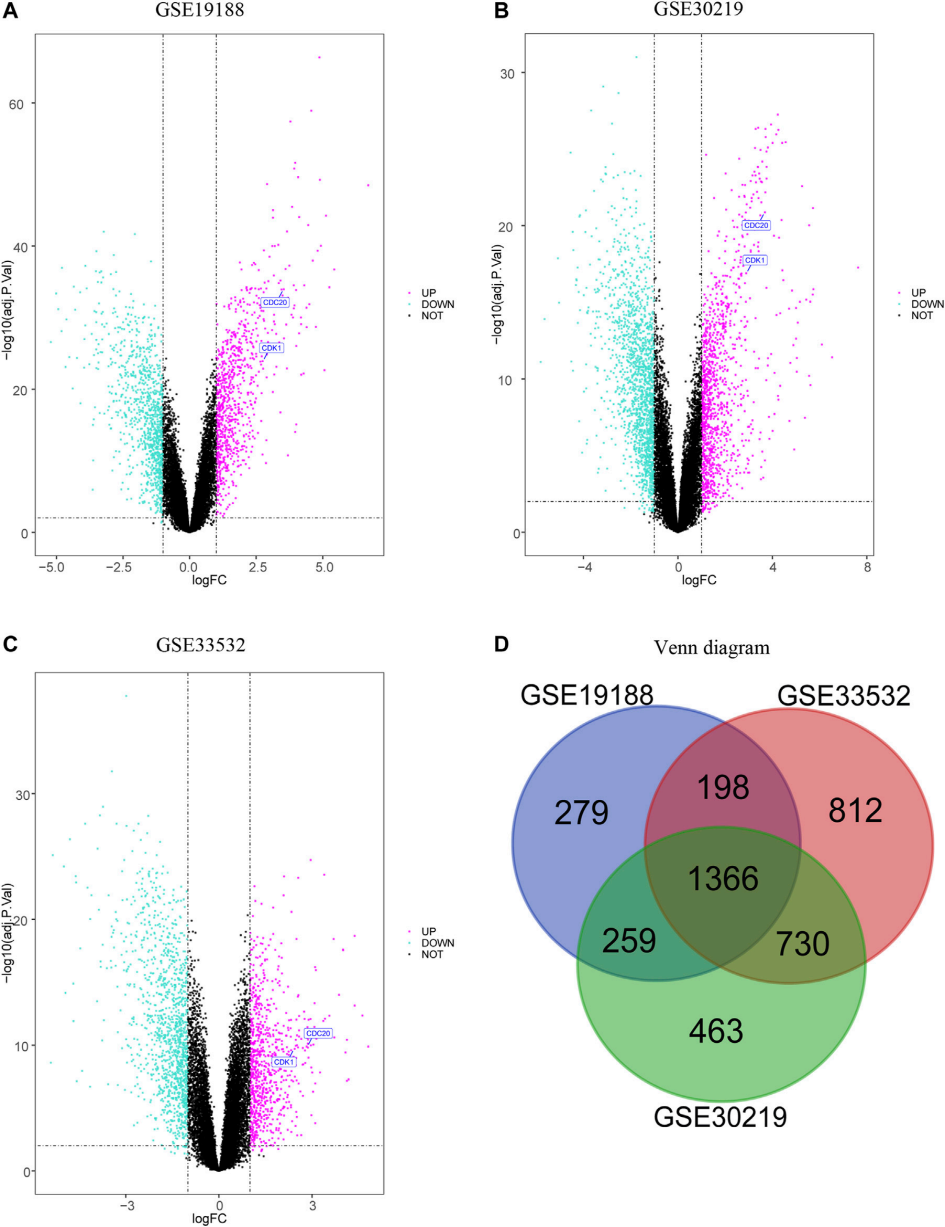
Volcano plot and Venn diagram of three expression profiles. The selection procession of downregulated and upregulated genes in GSE19188 **(A)**, GSE30219 **(B)** and GSE33532 **(C)** with *p*-value<0.05 and |logFC| > 1, and upregulated genes are marked in red, and downregulated genes are marked in green. The three datasets display an overlap of 1366 DEG **(D)**.

The volcano plots reveal the downregulated and upregulated genes in GSE19188 (Figure 1A), GSE30219 (Figure 1B), and GSE33532 (Figure 1C). After the normalization of the microarray outcomes, DEGs are selected in LUSC and normal lung tissue samples. The overlap among the three datasets consists of 1,366 genes (Figure 1D), including 669 downregulated and 697 upregulated genes. Fifty upregulated and fifty downregulated genes of the three datasets are indicated in heatmaps (Supplememtary Figure 1).

### Functional Enrichment Analysis

**FIGURE 2:**
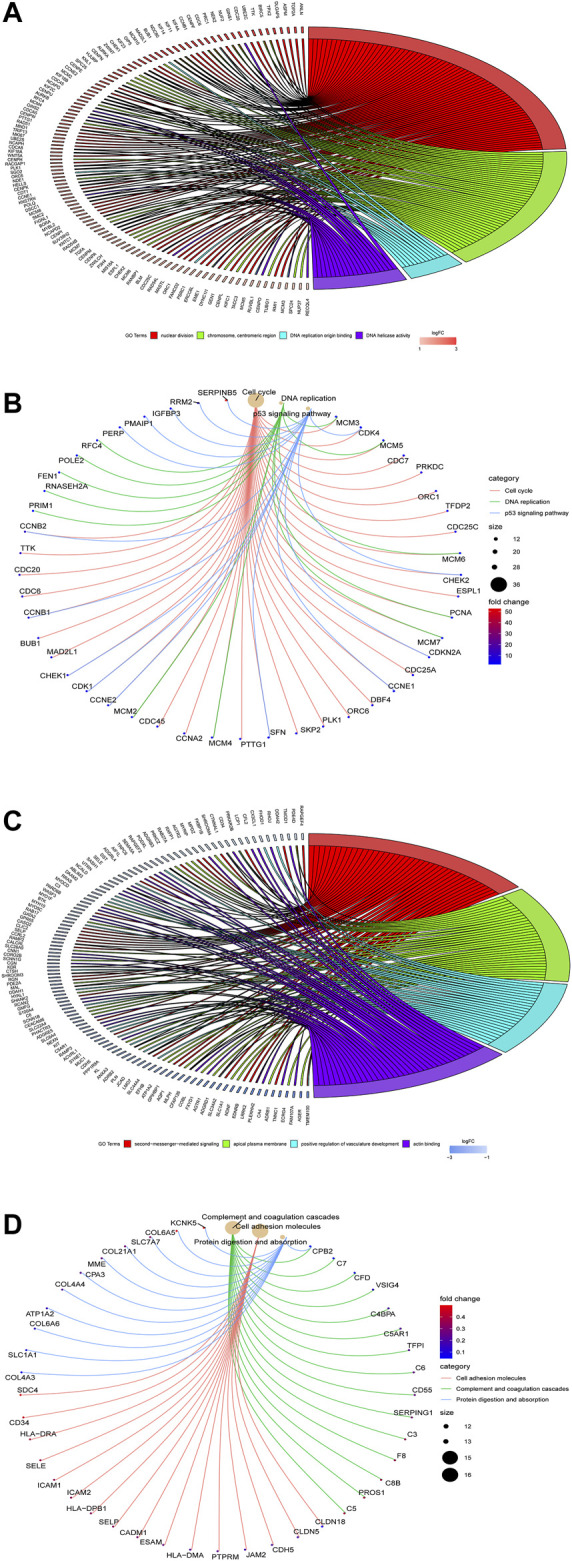
Go enrichment analysis of the DEGs using the clusterProfiler and GOplot packages. GO enrichment **(A)** and KEGG pathway **(B)** analyses of upregulated DEGs are performed, while GO enrichment **(C)** and KEGG pathway **(D)** analyses of downregulated DEGs are performed.

The results of the GO enrichment analysis of DEGs are illustrated as GO plots using the GOplot package. GO enrichment analyses of upregulated DEGs demonstrate that nuclear division, chromosome centromeric region, and DNA replication origin binding are primarily enriched in MF (Figure 2A), while KEGG pathway analysis indicates that cell cycle, DNA replication, and p53 signaling pathway are mainly enriched (Figure 2B), suggesting that upregulated genes are highly associated with cell cycle. In addition, GO enrichment analyses of downregulated DEGs suggest that second-messenger-medicated signaling, apical part of cell, and positive regulation of vasculature development are significantly enriched in MF (Figure 2C), whereas KEGG pathway analysis manifests that cell adhesion, complement and coagulation cascades, and protein digestion and absorption are primarily enriched (Figure 2D).

### Construction of the PPI Network and Selection of Hub Genes

**FIGURE 3:**
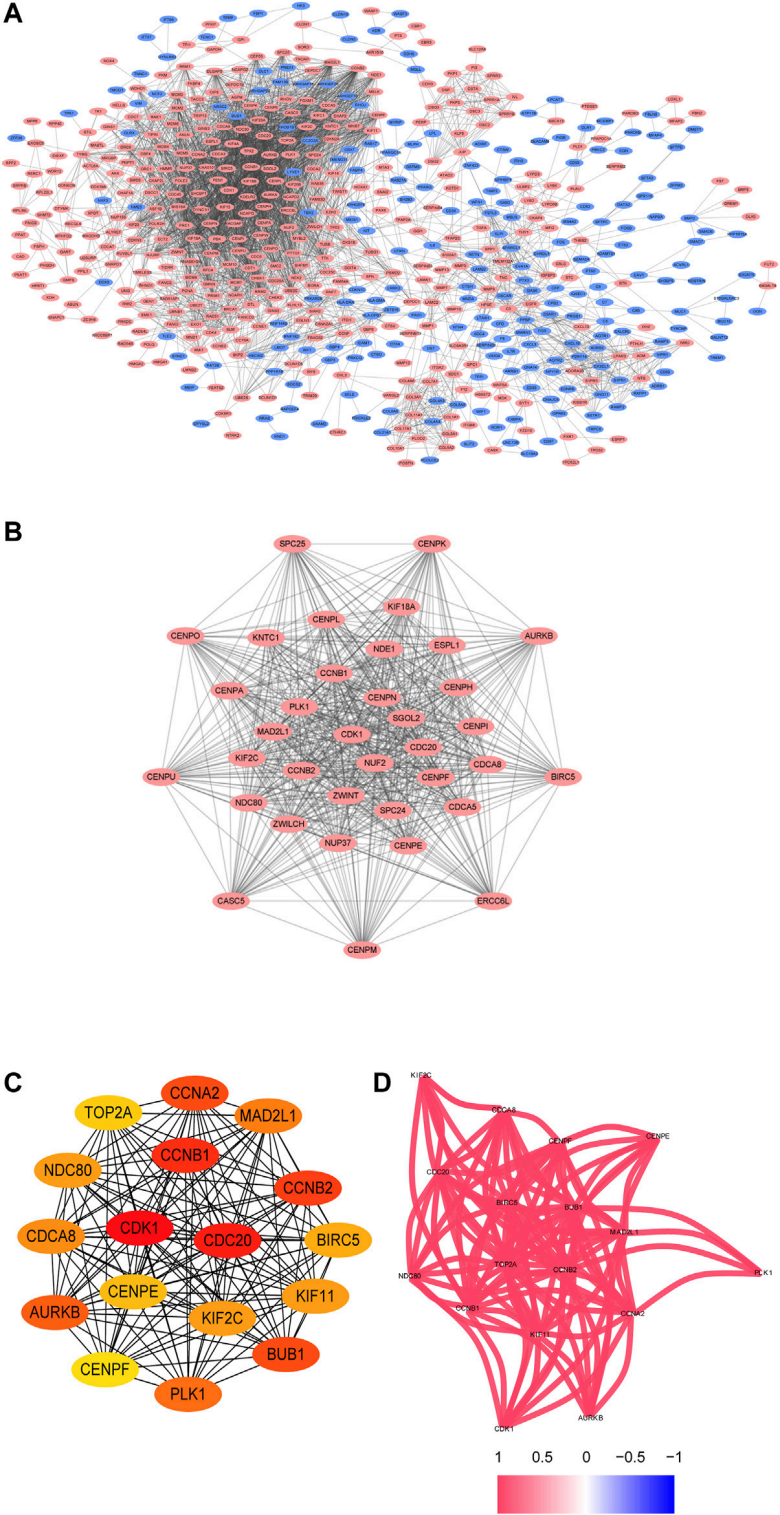
The PPI network and the two most significant modules of DEGs. The PPI network of DEGs is constructed using Cytoscape **(A)**. The most significant module is acquired from the PPI network with 37 nodes and 665 edges **(B)**. 17 hub genes are shown **(C)**. The correlation network of the hub gene is illustrated **(D)**. Upregulated genes are marked in red, and downregulated genes are marked in blue.

**FIGURE 4:**
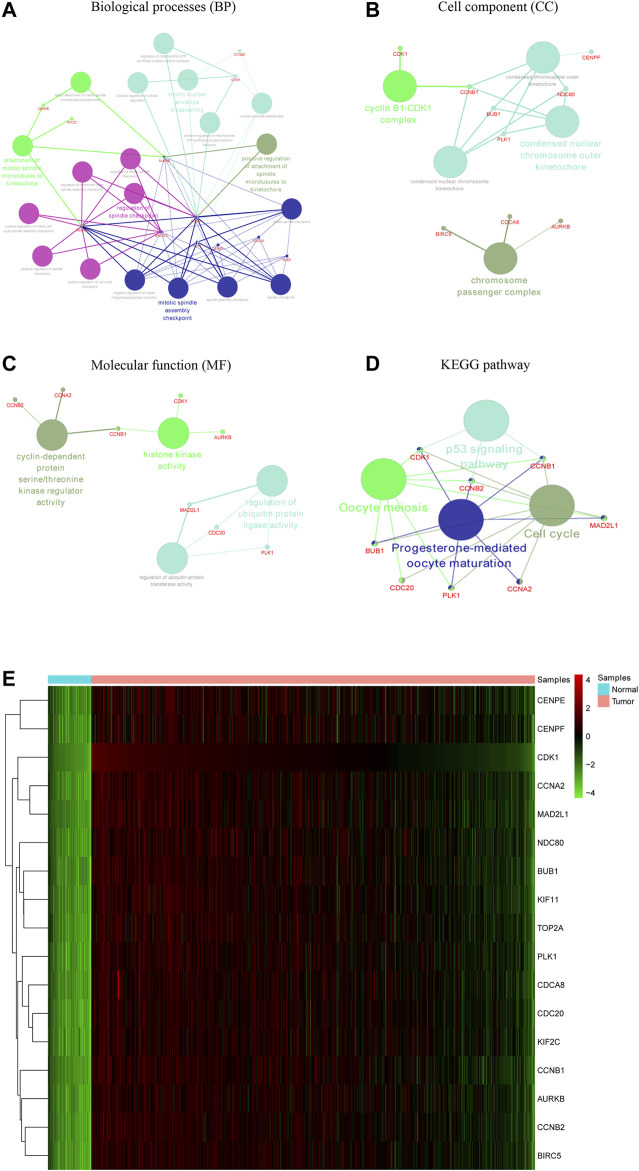
Functional enrichment analysis and heat map of these hub genes. The biological processes (BP), cell component (CC), and molecular function (MF) analysis of hub genes are performed using ClueGO **(A–C)**. The KEGG pathway analysis of hub genes is performed using ClueGO **(D)**. The two terms are marked in the same colors if they are similar, and the size of nodes refers to the *p*-value of biological process analysis. The hub genes participating in these terms are shown, and they are connected with relevant terms by solid lines. Heat map of hub genes is constructed based on data from TCGA and visualized using pheatmap package **(E)**. Up-regulation is marked in red, while down-regulation is marked in green.

The PPI network of the DEGs is displayed (Figure 3A) and includes 585 nodes and 3,797 edges. The most significant modules are identified via the MCODE plug-in. The most significant module includes 37 nodes and 665 edges (Figure 3B). Seventeen nodes are selected from the PPI network as the hub genes with degrees>60. 17 hub genes are all up-regulated genes, which are displayed (Figure 3C). Moreover, Table 3 lists gene symbols, degrees, full names, and functions of 17 hub genes. The correlation network indicates that these hub genes are closely correlated with each other. (Figure 3D). The GO enrichment analysis of 17 hub genes indicates that regulation of spindle checkpoint, mitotic spindle assembly checkpoint, condensed nuclear chromosome outer kinetochore, and cyclin-dependent protein serine/threonine kinase regulator activity are significantly enriched (Figures 4A–C). The KEGG pathway analysis of the hub genes indicates that cell cycle, p53 signaling pathway, and progesterone-mediated oocyte maturation are predominantly enriched (Figure 4D), revealing that these hub genes are closely correlated with cell cycle. Based on TCGA database, the heatmap of 17 hub genes suggests that all hub genes are expressed at high levels in the LUSC samples and expressed at low levels in the normal lung samples (Figure 4E).

**TABLE 3:** Functional roles of all hub genes with degrees>60.

No	Gene Symbol	Degree	Full name	Function
1	CDK1	123	Cyclin-dependent kinase 1	CDK1 regulates the cell cycle progression, apoptosis and carcinogenesis of several cancers
2	CDC20	106	Cell division cycle 20	CDC20 participates in the pathogenesis of NSCLC, and its higher expression correlates with higher tumor grade
3	CCNB1	95	Cyclin B1	CCNB1 plays an important role in promoting the transition from G2/M phase, and its high expression predicts worse prognosis in LUSC.
4	CCNB2	91	Cyclin B2	CCNB2 participates in promoting G2/M transition, and its overexpression correlates with the development of LUSC.
5	CCNA2	83	Cyclin A2	CCNA2 promotes the transition from G1 to S phase and G2 to M phase, and it is overexpressed in LUSC.
6	BUB1	83	BUB1 mitotic checkpoint serine/threonine kinase	BUB1 participates in binding kinetochores, and its mutation is correlated with the occurrence of LUSC.
7	AURKB	82	Aurora Kinase B	AURKB closely correlates with the acquired resistance of anti-EGFR treatment in NSCLC.
8	PLK1	80	Polo Like Kinase 1	Abnormal expression of PLK1 is associated with higher stages and aggressive progression of LUSC.
9	MAD2L1	75	Mitotic Arrest Deficient 2 Like 1	MAD2L1 participates in chromosomal instability and substantial aneuploidy, which is correlated with the carcinogenesis of NSCLC.
10	CDCA8	74	Cell Division Cycle Associated 8	Phosphorylation and activation of CDCA8 by AURKB serve an important role in the tumorigenesis of LC.
11	KIF11	69	Kinesin Family Member 11	The overexpression of KIF11 promotes the invasion and adverse prognosis of NSCLC.
12	KIF2C	69	Kinesin Family Member 2C	Elevated expression of KIF2C is associated with poorer differentiation status and lymph node metastasis in NSCLC.
13	NDC80	69	NDC80 Kinetochore Complex Component	NDC80 is highly expressed in LUAD, and high expression of NDC80 is associated with shorter OS.
14	BIRC5	66	Baculoviral IAP Repeat Containing 5	BIRC5 is highly expressed in LUSC, and the upregulation of BIRC5 indicates worse survival in LUSC.
15	CENPE	65	Centromere Protein E	CENPE is overexpressed in the G2/M phase, and CENPE participates in the cell proliferation in LUAD.
16	TOP2A	64	Topoisomerase (DNA) II α	TOP2A regulates the progress of LUSC, and high expression of TOP2A predicts poor prognosis in LUSC.
17	CENPF	63	Centromere Protein F	High expression of CENPF is associated with larger tumor size and poorer survival in NSCLC.

Abbreviations: LC, lung cancer; NSCLC, non-small-cell lung cancer; LUAD, lung adenocarcinoma; LUSC, lung squamous cell carcinoma.

**FIGURE 5:**
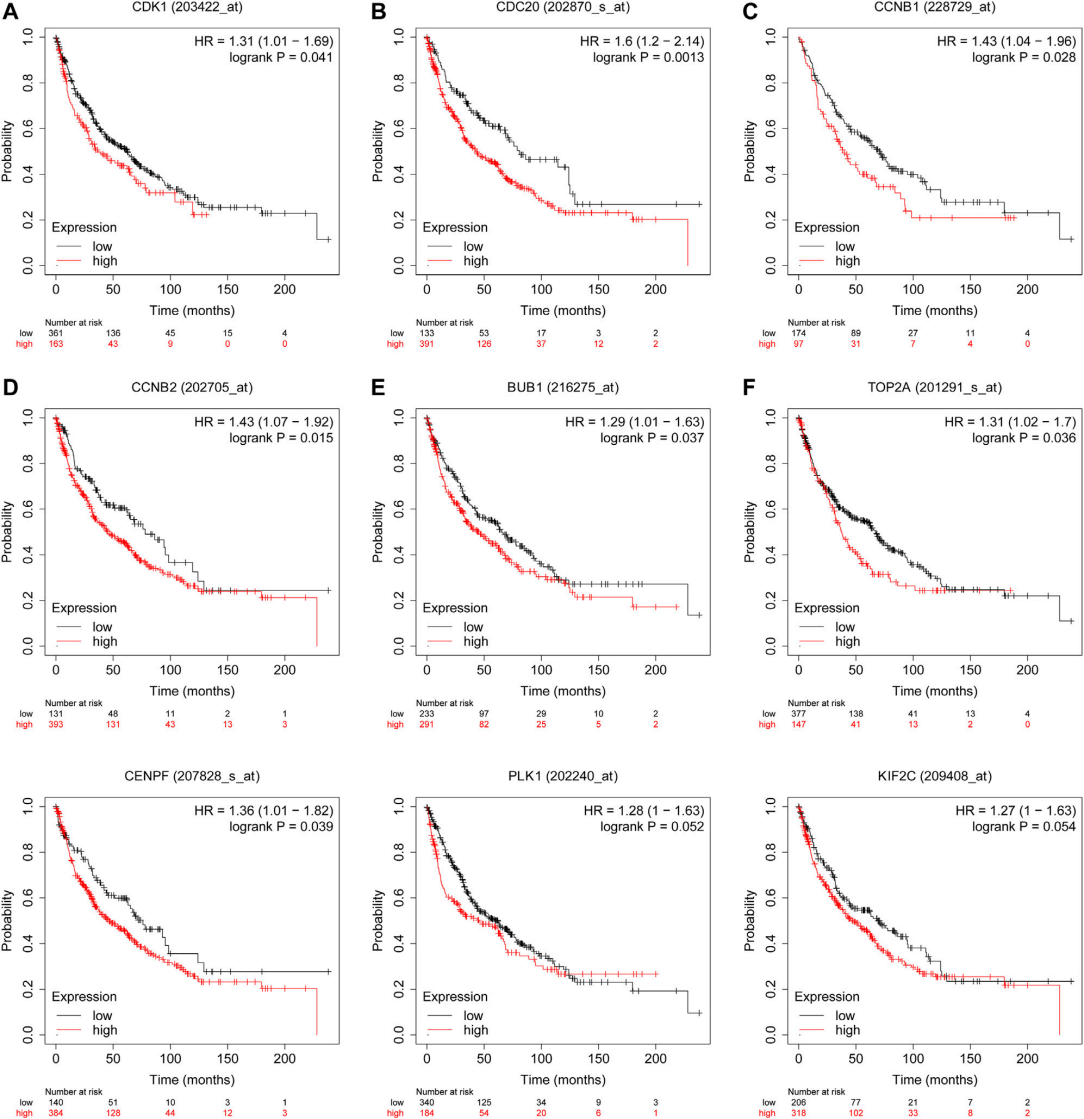
Overall survival analyses of hub genes are performed in Kaplan-Meier plotter online platform. CDK1, CDC20, CCNB1, CCNB2, BUB1, TOP2A, and CENPF **(A–G)** are negatively associated with OS. Prognostic values of PLK1 and KIF2C **(H,I)** are explored in LUSC patients. *p* < 0.05 is considered statistically significant.

Survival analyses indicated that CDK1, CDC20, CCNB1, CCNB2, BUB1, TOP2A, and CENPF are associated with worse OS in LUSC patients (Figure 5). In detail, Supplementary Table 2 lists the median OS, hazard ratio (HR), 95% confidence interval (CI), and log-rank P-value of these hub genes. CDK1 and CDC20 have the highest node degree scores (123 and 106, respectively), and both of them are closely associated with cell cycle, indicating that the two genes may serve core roles in the occurrence and progression of LUSC. Our survival analyses suggest that high expression levels of CDK1 and CDC20 are correlated with poor OS (*p* = 0.041 and *p* = 0.0013, respectively).

### Gene Set Enrichment Analysis of CDK1 and CDC20

**FIGURE 6:**
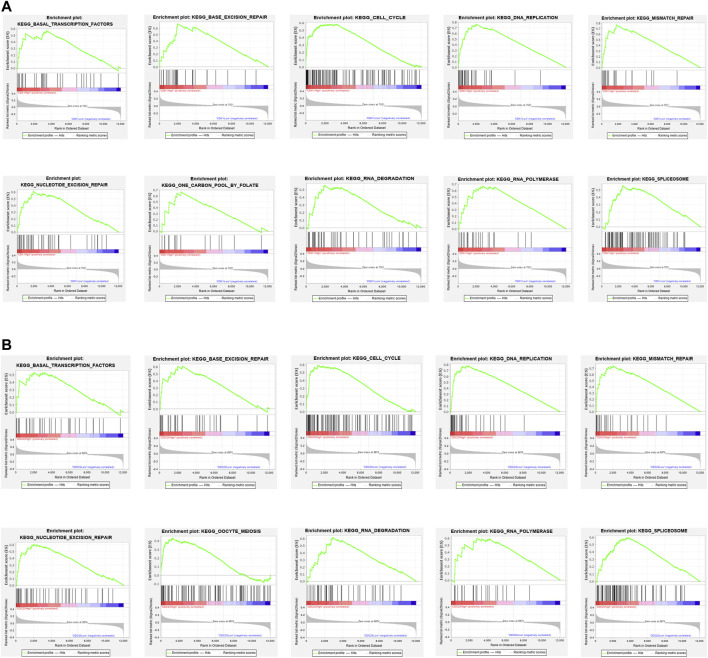
Enrichment plots by GSEA. Relative pathways associated with the expression of CDK1 **(A)** are displayed. Relative pathways associated with the expression of CDC20 **(B)** are displayed.

The results of the GSEA analysis indicate that numerous pathways, such as cell cycle, DNA replication, mismatch repair, and base excision repair, are associated with CDK1 expression (Figure 6A). The results of the GSEA analysis indicate that multiple pathways, containing DNA replication, cell cycle, mismatch repair, and RNA degradation are correlated with CDC20 expression (Figure 6B). The detailed information of the GSEA analysis of CDK1 and CDC20 is listed in Table 4.

**TABLE 4:** Relative pathways associated with the expression of CDK1 and CDC20 using GSEA.

Gene	Name	ES	NES	NOM *p*-value	FDR *q*-value
CDK1	KEGG_BASAL_TRANSCRIPTION_FACTORS	0.57	1.74	0.010	0.033
	KEGG_BASE_EXCISION_REPAIR	0.67	1.90	0.000	0.040
	KEGG_CELL_CYCLE	0.58	1.88	0.000	0.012
	KEGG_DNA_REPLICATION	0.76	1.85	0.004	0.014
	KEGG_MISMATCH_REPAIR	0.77	1.94	0.002	0.016
	KEGG_NUCLEOTIDE_EXCISION_REPAIR	0.61	1.81	0.006	0.017
	KEGG_ONE_CARBON_POOL_BY_FOLATE	0.67	1.73	0.002	0.034
	KEGG_RNA_DEGRADATION	0.56	1.83	0.006	0.016
	KEGG_RNA_POLYMERASE	0.67	1.88	0.000	0.015
	KEGG_SPLICEOSOME	0.55	1.95	0.002	0.020
CDC20	KEGG_BASAL_TRANSCRIPTION_FACTORS	0.53	1.57	0.033	0.137
	KEGG_BASE_EXCISION_REPAIR	0.61	1.70	0.004	0.063
	KEGG_CELL_CYCLE	0.59	1.84	0.002	0.030
	KEGG_DNA_REPLICATION	0.78	1.76	0.000	0.044
	KEGG_MISMATCH_REPAIR	0.74	1.80	0.004	0.034
	KEGG_NUCLEOTIDE_EXCISION_REPAIR	0.61	1.72	0.006	0.058
	KEGG_OOCYTE_MEIOSIS	0.43	1.60	0.011	0.126
	KEGG_RNA_DEGRADATION	0.62	1.94	0.000	0.010
	KEGG_RNA_POLYMERASE	0.60	1.60	0.031	0.134
	KEGG_SPLICEOSOME	0.59	2.03	0.002	0.005

Abbreviations: FDR, false discovery rate; GSEA, gene set enrichment analysis; NES, normalized enrichment score; NOM, nominal.

### The Verification Based on Public Databases

**FIGURE 7:**
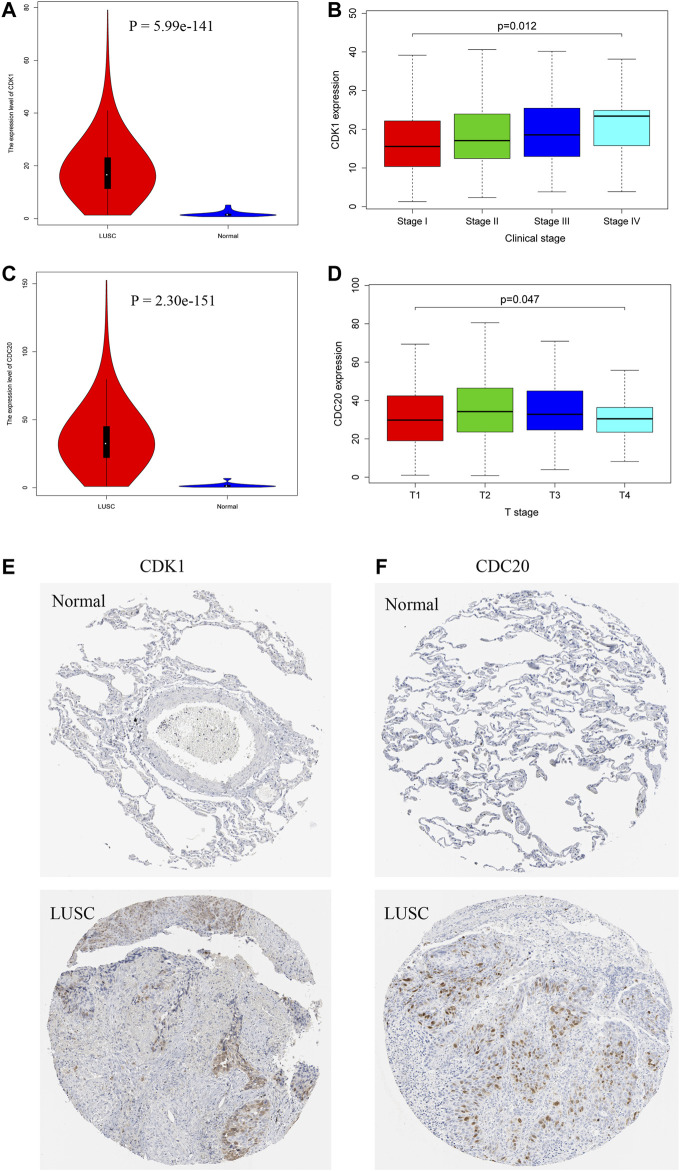
The expression levels of hub genes in LUSC based on TCGA and THPA databases. CDK1 **(A)** and CDC20 **(C)** are obviously upregulated in LUSC tissues. CDK1 is significantly associated with clinical stages **(B)**, and CDC20 is significantly correlated with T stages **(D)**. Immunohistochemistry (IHC) results reveal that CDK1 **(E)** and CDC20 **(F)** are overexpressed in LUSC tissues compared with normal tissues.

The expression level of CDK1 is significantly increased in 502 LUSC tissue samples compared with that in 49 normal tissue samples (Figure 7A). The CDK1 mRNA expression in LUSC is associated with the severity of the clinical stages, and the highest mRNA expression is observed in stage IV (Figure 7B). CDC20 is expressed at high levels in LUSC tissue samples compared that in normal tissue samples (Figure 7C). The CDC20 mRNA expression is correlated with the severity of the T stages (Figure 7D). Moreover, IHC results from the THPA database confirm that the protein expression levels of CDK1 and CDC20 are consistent with the results of the mRNA expression; namely, CDK1 and CDC20 are significantly upregulated in LUSC (Figures 7E,F). The detailed results of IHC of CDK1 and CDC20 are illustrated in Supplementary Table 3.

### The External Validation of Basic Experiments

**FIGURE 8:**
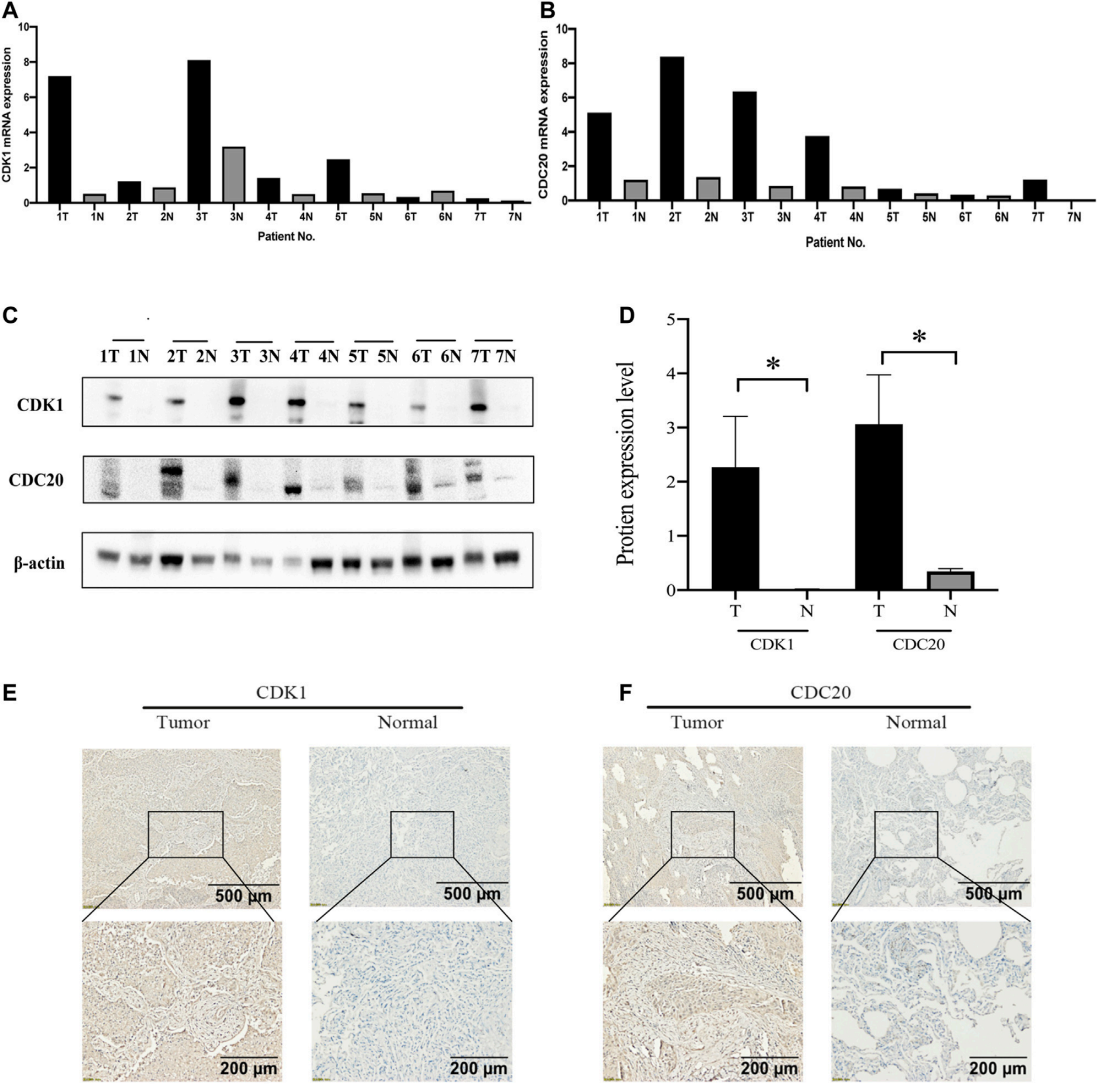
The validation of basic experiments. Real time-qPCR manifests that CDK1 **(A)** and CDC20 **(B)** in LUSC tissues are higher than normal lung tissues at the mRNA expression level. Western blot reveals that CDK1 and CDC20 in LUSC tissues are obviously higher than normal lung tissues at the protein expression level **(C,D)**. Immunohistochemistry (IHC) results reveal that CDK1 **(E)** and CDC20 **(F)** are overexpressed in LUSC tissues compared with normal tissues.

The mRNA expression levels of CDK1 and CDC20 in seven pairs of randomly selected tissues are showed, and CDK1 and CDC20 are significantly higher compared with surrounding normal tissues (Figures 8A,B). Also, we investigate the distribution and expression of CDK1 and CDC20 proteins in the 7 pairs of randomly selected tissues. Western blot analysis is highly consistent with the outcomes of mRNA levels, namely, CDK1 and CDC20 are highly expressed in LUSC than those in normal lung tissues (*p* < 0.05; Figures 8C,D). The representative image shows that CDK1 and CDC20 protein are mainly distributed in the cytoplasm/membrane, and both have high expression in LUSC (Figures 8E,F).

## Discussion

Three gene expression profiles were analyzed in the present study, and 1,366 DEGs were acquired, including 669 downregulated and 697 upregulated genes. These DEGs are mainly enriched in several pathways correlated with cell cycle, including nuclear division, cell cycle, and DNA replication. Through further selection, 17 hub genes were selected. Heatmaps indicate these hub genes are upregulated in the LUSC tissues. CDK1 and CDC20 are found to be survival-related hub genes. Public databases and basic experiments confirm that CDK1 and CDC20 are upregulated in LUSC at mRNA and protein levels.

CDK1 promotes cell entry into mitosis by binding to cyclin B to form cyclin B-CDK1 (Hiraoka et al., 2019). CDK1 is upregulated in various cancers, including LUSC, LUAD, and liver cancer (Stauffer et al., 2017). Many studies reported the important role of CDK1 in the malignancy of lung cancer. Singh et al. (2007) demonstrated that HEI10 negatively regulates cell motility and invasion by inhibiting cyclin B-CDK1 in tumor cells (Singh et al., 2007). Long noncoding RNA (lncRNA) CASC11 contributes to carcinogenesis and progression of lung cancer by targeting the miRNA-302/CDK1 axis (Tong et al., 2019). Similarly, lncRNA OR3A4 is involved in apoptosis, cell cycle, and cisplatin resistance by upregulating CDK1 in NSCLC (Shang et al., 2019). Autocrine IL-10 regulates the Op18/stathmin signaling via the IL-10-NF-κB-ERK/CDK1 axis to initiate carcinogenesis in NSCLC (Zhao et al., 2019). Furthermore, some studies demonstrated that CDK1 may be a therapeutic target for NSCLC. Iron-dependent CDK1 contributes to tumorigenesis in lung cancer via the GP130/STAT3 axis, and the inhibition of CDK1 reduces carcinogenicity of the tumor cells suggesting the therapeutic potential of CDK1 in lung cancer (Kuang et al., 2019). Chen et al. suggested that the purvalanol A (CDK1 inhibitor) can increase the cytotoxicity of paclitaxel via Op18/stathmin in NSCLC (Chen et al., 2017). Shi et al. demonstrated that miRNA-181a suppresses cell proliferation by targeting CDK1 in NSCLC (Shi et al., 2017). Moreover, CDK1 may be used to predict prognosis because high expression of CDK1 is associated with a worse prognosis in NSCLC (Liu et al., 2018). The results of survival analysis in this study are consistent with their findings.

CDC20 encodes an important spindle assembly checkpoint (SAC) protein that can activate the anaphase-promoting complex (APC/C) (Fujimitsu and Yamano, 2020). A mistake during the segregation of sister chromatids prevents mitotic arrest due to abnormal levels of CDC20 thus contributing to premature anaphase and causing aneuploidy, which is associated with malignant transformation of the tumor cells (Lara-Gonzalez et al., 2019). The elevated expression of CDC20 is often observed in cancer, including NSCLC and breast cancer (Li et al., 2019). Numerous studies demonstrated that CDC20 plays an important role in the development of NSCLC. CDC20 participates in the pathogenesis of NSCLC, and elevated expression of CDC20 is associated with higher tumor grade and stage (Gayyed et al., 2016). Zhang Y et al. demonstrated that CDC20 is involved in the occurrence and progression of NSCLC (Zhang et al., 2015). Additionally, several studies have reported the associations between CDC20 and prognosis in NSCLC. The abnormal expression of CDC20 is closely associated with worse survival in NSCLC patients (Zhang et al., 2015). Similarly, Kato et al. (2012) demonstrated that elevated expression of CDC20 correlates with pleural invasion and inferior 5-year OS in 362 NSCLC cases (Kato et al., 2012). The results of the survival analysis in the present study confirm the associations of CDC20. CDC20 is a potential target for the treatment of cancer. Knockdown of CDC20 via small interfering RNA (siRNA) inhibits the growth of the tumor cells, leads to the accumulation of the cells in the G2/M-phase, and improves the cytotoxicity of paclitaxel (Taniguchi et al., 2008). External validation suggests that CDK1 and CDC20 are highly expressed in LUSC through public databases and basic experiments. Thus, CDK1 and CDC20 may play important roles in carcinogenesis and development of LUSC and may be the candidate therapeutic targets and prognostic biomarkers in LUSC.

Several limitations in our study are listed as follows. First, only three datasets are included in our analysis. Although some datasets are similar, they fail to meet the selection criteria described above. To reduce the bias of our study, we excluded these datasets. Additional relevant investigations are needed to further elucidate the role of CDK1 and CDC20 in LUSC. Second, the included datasets fail to provide detailed survival information, and we had to perform the overall survival analysis of the hub genes using the K-M plotter.

## Conclusion

Our study was performed to identify DEGs associated with the tumorigenesis and invasion of LUSC. Seventeen hub genes were selected from 1,366 DEGs, and CDK1 and CDC20 were shown to be correlated with the prognosis of LUSC. Furthermore, public databases and basic experiments validated the upregulated status of CDK1 and CDC20. CDK1 and CDC20 may be potential therapeutic and prognostic indicators of LUSC. Additional studies are demanded to investigate the biological associations of the two genes with LUSC.

## Data Availability

The original contributions presented in the study are included in the article/Supplementary Material, further inquiries can be directed to the corresponding authors.

## Abbreviations

ALK, anaplastic lymphoma kinase; BP, biological processes; CC, cell component; CI, confidence interval; DEGs, differentially expressed genes; EGFR, epidermal growth factor receptor; FC, fold change; GEO, gene expression omnibus; GO, gene ontology; GSEA, gene set enrichment analysis; HR, hazard ratio; KEGG, kyoto encyclopedia of genes and genomes; K-M plotter, Kaplan Meier plotter; LUAD, lung adenocarcinoma; LUSC, lung squamous cell carcinoma; lncRNA, Long non-coding RNA; MCODE, Molecular Complex Detection; MET, MET Proto-Oncogene; MF, molecular function; MSigDB, molecular signatures database; NSCLC, non-small cell lung cancer; OS, overall survival; PIK3CA, Phosphatidylinositol-4,5- Bisphosphate 3-Kinase Catalytic Subunit Alpha; PPI, protein-protein interaction; siRNA, small interfering RNA; STRING, Search Tool for the Retrieval of Interacting Genes; TCGA, The Cancer Genome Atlas; THPA, The Human Protein Altas; RT-qPCR, real time-quantitative PCR.

## References

[B1] Ashburner M. Ball C. A. Blake J. A. Botstein D. Butler H. Cherry J. M. (2000). Gene Ontology: Tool for the Unification of Biology. Nat. Genet. 25 (1), 25–29. 10.1038/75556 10802651 PMC3037419

[B2] Bandettini W. P. Kellman P. Mancini C. Booker O. J. Vasu S. Leung S. W. (2012). MultiContrast Delayed Enhancement (MCODE) Improves Detection of Subendocardial Myocardial Infarction by Late Gadolinium Enhancement Cardiovascular Magnetic Resonance: a Clinical Validation Study. J. Cardiovasc. Magn. Reson. 14, 83. 10.1093/nar/gks1094 23199362 PMC3552709

[B3] Barrett T. Wilhite S. E. Ledoux P. Evangelista C. Kim I. F. Tomashevsky M. (2013). NCBI GEO: Archive for Functional Genomics Data Sets-Uupdate. Nucleic Acids Res. 41 (Database issue), D991–D995. 10.1093/nar/gks1193 23193258 PMC3531084

[B4] Chen X. Liao Y. Long D. Yu T. Shen F. Lin X. (2017). The Cdc2/Cdk1 Inhibitor, Purvalanol A, Enhances the Cytotoxic Effects of Taxol through Op18/stathmin in Non-small Cell Lung Cancer Cells *In Vitro* . Int. J. Mol. Med. 40 (1), 235–242. 10.3892/ijmm.2017.2989 28534969

[B5] Chin C.-H. Chen S.-H. Wu H.-H. Ho C.-W. Ko M.-T. Lin C.-Y. (2014). cytoHubba: Identifying Hub Objects and Sub-networks from Complex Interactome. BMC Syst. Biol. 8 (Suppl. 4), S11. 10.1186/1752-0509-8-s4-s11 25521941 PMC4290687

[B6] Dawany N. B. Dampier W. N. Tozeren A. (2011). Large-scale Integration of Microarray Data Reveals Genes and Pathways Common to Multiple Cancer Types. Int. J. Cancer 128 (12), 2881–2891. 10.1002/ijc.25854 21165954

[B7] Franceschini A. Szklarczyk D. Frankild S. Kuhn M. Simonovic M. Roth A. (2013). STRING v9.1: Protein-Protein Interaction Networks, with Increased Coverage and Integration. Nucleic Acids Res. 41 (Database issue), D808–D815. 10.1093/nar/gks1094 23203871 PMC3531103

[B8] Fujimitsu K. Yamano H. (2020). PP2A-B56 Binds to Apc1 and Promotes Cdc20 Association with the APC/C Ubiquitin Ligase in Mitosis. EMBO Rep. 21 (1), e48503. 10.15252/embr.201948503 31825153 PMC6945068

[B9] Gayyed M. F. El-Maqsoud N. M. R. A. Tawfiek E. R. El Gelany S. A. A. Rahman M. F. A. (2016). A Comprehensive Analysis of CDC20 Overexpression in Common Malignant Tumors from Multiple Organs: its Correlation with Tumor Grade and Stage. Tumor Biol. 37, 749–762. 10.1007/s13277-015-3808-1 26245990

[B10] Győrffy B. Surowiak P. Budczies J. Lánczky A. (2013). Online Survival Analysis Software to Assess the Prognostic Value of Biomarkers Using Transcriptomic Data in Non-small-cell Lung Cancer. PLoS One 8 (12), e82241. 10.1371/journal.pone.0082241 24367507 PMC3867325

[B11] Hiraoka D. Hosoda E. Chiba K. Kishimoto T. (2019). SGK Phosphorylates Cdc25 and Myt1 to Trigger Cyclin B-Cdk1 Activation at the Meiotic G2/M Transition. J. Cel Biol 218 (11), 3597–3611. 10.1083/jcb.201812122 PMC6829662 31537708

[B12] Hou J. Aerts J. den Hamer B. Ijcken W. V. Bakker M. D. Riegman P. (2010). Gene Expression-Based Classification of Non-small Cell Lung Carcinomas and Survival Prediction. PLoS One 5 (4), e10312. 10.1126/scitranslmed.3005723 20421987 PMC2858668

[B13] Kanehisa M. Furumichi M. Tanabe M. Sato Y. Morishima K. (2017). KEGG: New Perspectives on Genomes, Pathways, Diseases and Drugs. Nucleic Acids Res. 45 (D1), D353–D361. 10.1093/nar/gkw1092 27899662 PMC5210567

[B14] Kato T. Daigo Y. Aragaki M. Ishikawa K. Sato M. Kaji M. (2012). Overexpression of CDC20 Predicts Poor Prognosis in Primary Non-small Cell Lung Cancer Patients. J. Surg. Oncol. 106 (4), 423–430. 10.1002/jso.23109 22488197

[B15] Kuang Y. Guo W. Ling J. Xu D. Liao Y. Zhao H. (2019). Iron-dependent CDK1 Activity Promotes Lung Carcinogenesis via Activation of the GP130/STAT3 Signaling Pathway. Cell Death Dis 10 (4), 297. 10.1038/s41419-019-1528-y 30931929 PMC6443808

[B16] Lara-Gonzalez P. Moyle M. W. Budrewicz J. Mendoza-Lopez J. Oegema K. Desai A. (2019). The G2-To-M Transition Is Ensured by a Dual Mechanism that Protects Cyclin B from Degradation by Cdc20-Activated APC/C. Develop. Cel 51 (3), 313–325.e10. 10.1016/j.devcel.2019.09.005 PMC7778526 31588029

[B17] Li J. Dang N. Martinez-Lopez N. Jowsey P. A. Huang D. Lightowlers R. N. (2019). M2I-1 Disrupts the *In Vivo* Interaction between CDC20 and MAD2 and Increases the Sensitivities of Cancer Cell Lines to Anti-mitotic Drugs via MCL-1s. Cell Div 14, 5. 10.1186/s13008-019-0049-5 31249607 PMC6570884

[B18] Li S. Choi Y.-L. Gong Z. Liu X. Lira M. Kan Z. (2016). Comprehensive Characterization of Oncogenic Drivers in Asian Lung Adenocarcinoma. J. Thorac. Oncol. 11 (12), 2129–2140. 10.1016/j.jtho.2016.08.142 27615396

[B19] Liberzon A. Subramanian A. Pinchback R. Thorvaldsdottir H. Tamayo P. Mesirov J. P. (2011). Molecular Signatures Database (MSigDB) 3.0. Bioinformatics 27 (12), 1739–1740. 10.1093/bioinformatics/btr260 21546393 PMC3106198

[B20] Liu W.-T. Wang Y. Zhang J. Ye F. Huang X.-H. Li B. (2018). A Novel Strategy of Integrated Microarray Analysis Identifies CENPA, CDK1 and CDC20 as a Cluster of Diagnostic Biomarkers in Lung Adenocarcinoma. Cancer Lett. 425, 43–53. 10.1016/j.canlet.2018.03.043 29608985

[B21] Manoli T. Gretz N. Gröne H.-J. Kenzelmann M. Eils R. Brors B. (2006). Group Testing for Pathway Analysis Improves Comparability of Different Microarray Datasets. Bioinformatics 22 (20), 2500–2506. 10.1093/bioinformatics/btl424 16895928

[B22] Meister M. Belousov A. Xu E. C. (2014). Intra-tumor Heterogeneity of Gene Expression Profiles in Early Stage Non-small Cell Lung Cancer. J. Bioinformatics Res. Stud. 1 (1), 1.

[B23] Mlecnik B. Galon J. Bindea G. (2019). Automated Exploration of Gene Ontology Term and Pathway Networks with ClueGO-REST. Bioinformatics 35 (19), 3864–3866. 10.1093/bioinformatics/btz163 30847467 PMC6761950

[B24] Ritchie M. E. Phipson B. Wu D. Hu Y. Law C. W. Shi W. (2015). Limma powers Differential Expression Analyses for RNA-Sequencing and Microarray Studies. Nucleic Acids Res. 43 (7), e47. 10.1093/nar/gkv007 25605792 PMC4402510

[B25] Rousseaux S. Debernardi A. Jacquiau B. Vitte A. L. Vesin A. Nagy-Mignotte H. (2013). Ectopic Activation of Germline and Placental Genes Identifies Aggressive Metastasis-Prone Lung Cancers. Sci. Transl Med. 5 (186), 186ra66. 10.1126/scitranslmed.3005723 PMC4818008 23698379

[B26] Sands J. M. Nguyen T. Shivdasani P. Sacher A. G. Cheng M. L. Alden R. S. (2020). Next-generation Sequencing Informs Diagnosis and Identifies Unexpected Therapeutic Targets in Lung Squamous Cell Carcinomas. Lung Cancer 140, 35–41. 10.1016/j.lungcan.2019.12.005 31855703

[B27] Shang J. Xu Y. D. Zhang Y. Y. Li M. (2019). Long Noncoding RNA OR3A4 Promotes Cisplatin Resistance of Non-small Cell Lung Cancer by Upregulating CDK1. Eur. Rev. Med. Pharmacol. Sci. 23 (10), 4220–4225. 10.26355/eurrev_201905_17926 31173293

[B28] Shi Q. Zhou Z. Ye N. Chen Q. Zheng X. Fang M. (2017). MiR-181a Inhibits Non-small Cell Lung Cancer Cell Proliferation by Targeting CDK1. Cbm 20 (4), 539–546. 10.3233/cbm-170350 28946554

[B29] Siegel R. L. Miller K. D. Jemal A. (2020). Cancer Statistics, 2020. CA A. Cancer J. Clin. 70 (1), 7–30. 10.3322/caac.21590 31912902

[B30] Singh M. K. Nicolas E. Gherraby W. Dadke D. Lessin S. Golemis E. A. (2007). HEI10 Negatively Regulates Cell Invasion by Inhibiting Cyclin B/Cdk1 and Other Promotility Proteins. Oncogene 26 (33), 4825–4832. 10.1038/sj.onc.1210282 17297447 PMC2597433

[B31] Smoot M. E. Ono K. Ruscheinski J. Wang P. L. Ideker T. (2011). Cytoscape 2.8: New Features for Data Integration and Network Visualization. Bioinformatics 27 (3), 431–432. 21149340 10.1093/bioinformatics/btq675 PMC3031041

[B32] Stauffer S. Zeng Y. Zhou J. Chen X. Chen Y. Dong J. (2017). CDK1-mediated Mitotic Phosphorylation of PBK Is Involved in Cytokinesis and Inhibits its Oncogenic Activity. Cell Signal. 39, 74–83. 10.1016/j.cellsig.2017.08.001 28780319 PMC5592141

[B33] Subramanian A. Tamayo P. Mootha V. K. Mukherjee S. Ebert B. L. Gillette M. A. (2005). Gene Set Enrichment Analysis: a Knowledge-Based Approach for Interpreting Genome-wide Expression Profiles. Proc. Natl. Acad. Sci. 102 (43), 15545–15550. 10.1073/pnas.0506580102 16199517 PMC1239896

[B34] Taniguchi K. Momiyama N. Ueda M. Matsuyama R. Mori R. Fujii Y. (2008). Targeting of CDC20 via Small Interfering RNA Causes Enhancement of the Cytotoxicity of Chemoradiation. Anticancer Res. 28 (3A), 1559–1563. 18630511

[B35] Tong W. Han T. C. Wang W. Zhao J. (2019). LncRNA CASC11 Promotes the Development of Lung Cancer through Targeting microRNA-302/CDK1 axis. Eur. Rev. Med. Pharmacol. Sci. 23 (15), 6539–6547. 10.26355/eurrev_201908_18539 31378894

[B36] Yu G. Wang L.-G. Han Y. He Q.-Y. (2012). clusterProfiler: an R Package for Comparing Biological Themes Among Gene Clusters. OMICS: A J. Integr. Biol. 16 (5), 284–287. 10.1089/omi.2011.0118 PMC3339379 22455463

[B37] Zhang Y. Wang H. Wang J. Bao L. Wang L. Huo J. (2015). Global Analysis of Chromosome 1 Genes Among Patients with Lung Adenocarcinoma, Squamous Carcinoma, Large-Cell Carcinoma, Small-Cell Carcinoma, or Non-cancer. Cancer Metastasis Rev. 34 (2), 249–264. 10.1007/s10555-015-9558-0 25937073

[B38] Zhao Y. Chen S. Shen F. Long D. Yu T. Wu M. (2019). *In Vitro* neutralization of Autocrine IL 10 Affects Op18/stathmin Signaling in Nonsmall Cell Lung Cancer Cells. Oncol. Rep. 41 (1), 501–511. 10.3892/or.2018.6795 30320402

