# Supplementary material for: High Expression Levels of CDK1 and CDC20 in Patients With Lung Squamous Cell Carcinoma are Associated With Worse Prognosis
Source: Front Mol Biosci. 2021 Jul 7;8:653805. doi: 10.3389/fmolb.2021.653805 (PMC8292837; doi:10.3389/fmolb.2021.653805)
Supplement: Supplementary file 1 [file Table1.DOCX]

**Table S1** Major demographic and clinicopathological characteristics of LUSC patients (n=20)

| **Items** | **Value ^a^** |
| --- | --- |
| **Age (years)** | 70 (58-77) |
| **Sex**  Male  Female | 2 (10)  18 (90) |
| **Smoking status**  Yes  No | 17 (85)  3 (15) |
| **Tumor size (cms)**  $\leq$5  $>$5 | 10 (50)  10 (50) |
| **Differentiation**  Low  Moderate-low  Moderate  High-moderate | 5 (25)  3 (15)  10 (50)  2 (10) |
| **Lymph node metastasis**  Yes  No | 15 (75)  5 (25) |
| **Stage**  I  II  III | 2 (10)  2 (10)  16 (80) |
| **Tumor recurrence or metastasis**  Yes  No | 16 (80)  4 (20) |

LUSC: lung squamous cell carcinoma; TNM: tumor‑nodes‑metastasis. ^a^ values are expressed as median (range) or n (%).
