# Supplementary figures and images for: High Expression Levels of CDK1 and CDC20 in Patients With Lung Squamous Cell Carcinoma are Associated With Worse Prognosis
Source: Front Mol Biosci. 2021 Jul 7;8:653805. doi: 10.3389/fmolb.2021.653805 (PMC8292837; doi:10.3389/fmolb.2021.653805)

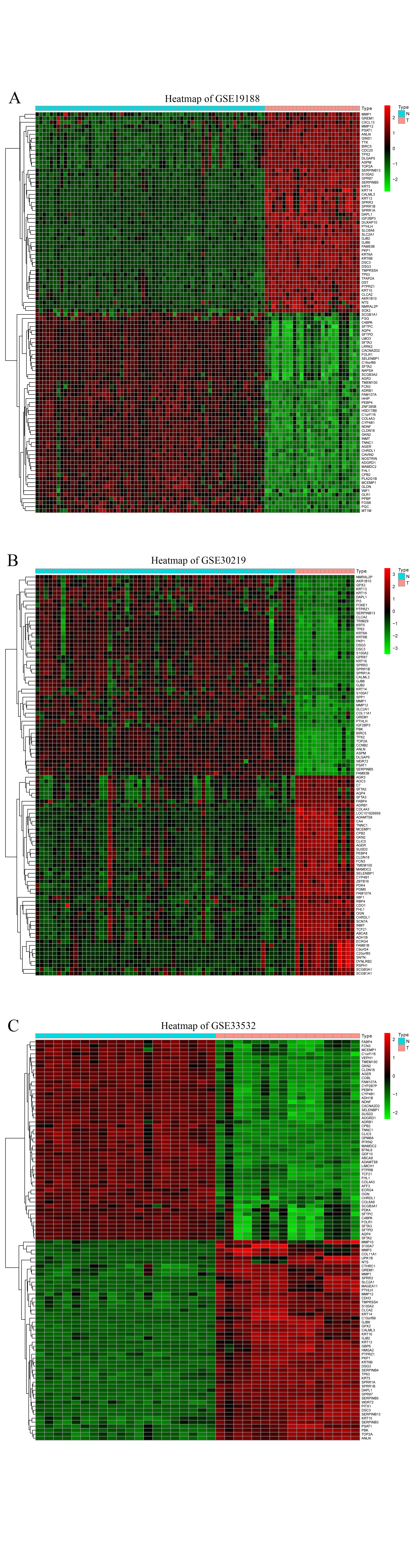

Supplement: Supplementary file 2 [file Image1.TIF]
