# Supplementary material for: High Expression Levels of CDK1 and CDC20 in Patients With Lung Squamous Cell Carcinoma are Associated With Worse Prognosis
Source: Front Mol Biosci. 2021 Jul 7;8:653805. doi: 10.3389/fmolb.2021.653805 (PMC8292837; doi:10.3389/fmolb.2021.653805)
Supplement: Supplementary file 3 [file Table2.DOCX]

**Table S2.** Overall survival analysis of all hub genes using K-M plotter.

| **Gene Symbol** | **HR** | **95%CI** | **Log-rank P-Value** |
| --- | --- | --- | --- |
| CDK1 | 1.31 | 1.01-1.69 | 0.041 |
| CDC20 | 1.60 | 1.20-2.14 | 0.0013 |
| CCNB1 | 1.43 | 1.04-1.96 | 0.028 |
| CCNB2 | 1.43 | 1.07-1.92 | 0.015 |
| CCNA2 | 0.90 | 0.70-1.16 | 0.42 |
| BUB1 | 1.29 | 1.01-1.63 | 0.037 |
| AURKB | 0.91 | 0.71-1.16 | 0.44 |
| PLK1 | 1.28 | 1.00-1.63 | 0.052 |
| MAD2L1 | 1.24 | 0.88-1.57 | 0.072 |
| CDCA8 | 1.13 | 0.87-1.49 | 0.36 |
| KIF11 | 1.24 | 0.93-1.66 | 0.15 |
| KIF2C | 1.27 | 1.00-1.63 | 0.054 |
| NDC80 | 1.28 | 0.98-1.67 | 0.071 |
| BIRC5 | 0.86 | 0.67-1.11 | 0.26 |
| CENPE | 1.16 | 0.91-1.47 | 0.23 |
| TOP2A | 1.31 | 1.02-1.70 | 0.036 |
| CENPF | 1.36 | 1.01-1.82 | 0.039 |

**Abbreviations:** K-M plotter: Kaplan‑Meier plotter; HR: hazard ratio; CI: confidence interval.
