# Supplementary material for: High Expression Levels of CDK1 and CDC20 in Patients With Lung Squamous Cell Carcinoma are Associated With Worse Prognosis
Source: Front Mol Biosci. 2021 Jul 7;8:653805. doi: 10.3389/fmolb.2021.653805 (PMC8292837; doi:10.3389/fmolb.2021.653805)
Supplement: Supplementary file 4 [file Table3.DOCX]

**Table S3** The detailed information of immunohistochemistry from The Human Protein Atlas database.

| **Gene** | | **Normal lung tissues** | | | | | | | |  | **LUSC tissues** | | | |  |
| --- | --- | --- | --- | --- | --- | --- | --- | --- | --- | --- | --- | --- | --- | --- | --- |
|  | | Macrophages | | |  | Pneumocytes | | | |  | Tumor cells | | | |  |
|  | Staining | Intensity | Quantity | Location |  | Staining | Intensity | Quantity | Location |  | Staining | Intensity | Quantity | Location |  |
| **CDK1** | Not detected | Negative | None | None |  | Not detected | Negative | None | None |  | Medium | Moderate | 75%-25% | Cytoplasmic/  membranous nuclear |  |
|  |  |  |  |  |  |  |  |  |  |  |  |  |  |  |  |
| **CDC20** | Not detected | Negative | None | None |  | Not detected | Negative | None | None |  | High | Strong | 75%-25% | Cytoplasmic/  membranous nuclear |  |
|  |  |  |  |  |  |  |  |  |  |  |  |  |  |  |  |

**Abbreviations:** LUSC: lung squamous cell carcinoma.
